# A novel MRI index for paraspinal muscle fatty infiltration: reliability and relation to pain and disability in lumbar spinal stenosis: results from a multicentre study

**DOI:** 10.1186/s41747-022-00284-y

**Published:** 2022-07-20

**Authors:** Hasan Banitalebi, Jørn Aaen, Kjersti Storheim, Anne Negård, Tor Åge Myklebust, Margreth Grotle, Christian Hellum, Ansgar Espeland, Masoud Anvar, Kari Indrekvam, Clemens Weber, Jens Ivar Brox, Helena Brisby, Erland Hermansen

**Affiliations:** 1 Department of Diagnostic Imaging, Akershus University Hospital, Lørenskog, Norway; 2 Institute of Clinical Medicine, University of Oslo, Oslo, Norway; 3 Department of Orthopaedic Surgery, Ålesund Hospital, Møre and Romsdal Hospital Trust, Ålesund, Norway; 4 Department of Circulation and Medical Imaging, Faculty of Medicine and Health Sciences, Norwegian University of Science and Technology, Trondheim, Norway; 5 Communication and Research Unit for Musculoskeletal Health (FORMI), Oslo University Hospital, Oslo, Norway; 6 Department of Research and Innovation, Møre and Romsdal Hospital Trust, Ålesund, Norway; 7 Department of Registration, Cancer Registry Norway, Oslo, Norway; 8 Department of Physiotherapy, Faculty of Health Science, Oslo Metropolitan University, P.O. box 4, St. Olafs plass, Oslo, Norway; 9 Division of Orthopaedic Surgery, Oslo University Hospital Ulleval, Oslo, Norway; 10 Department of Radiology, Haukeland University Hospital, Bergen, Norway; 11 Department of Clinical Medicine, University of Bergen, Bergen, Norway; 12 Unilabs Radiology, Oslo, Norway; 13 Kysthospitalet in Hagevik. Orthopaedic Clinic, Haukeland University Hospital, Bergen, Norway; 14 Department of Neurosurgery, Stavanger University Hospital, Stavanger, Norway; 15 Department of Quality and Health Technology, University of Stavanger, Stavanger, Norway; 16 Department of Physical Medicine and Rehabilitation, Oslo University Hospital, Oslo, Norway; 17 Department of Orthopaedics, Sahlgrenska University Hospital, Gothenburg, Sweden; 18 Department of Orthopaedics, Institute for clinical sciences, Sahlgrenska Academy, University of Gothenburg, Gothenburg, Sweden; 19 Hofseth BioCare, Ålesund, Norway

**Keywords:** Magnetic resonance imaging, Paraspinal muscles, Patient-reported outcome measures, Psoas muscles, Spinal stenosis

## Abstract

**Background:**

Fatty infiltration of the paraspinal muscles may play a role in pain and disability in lumbar spinal stenosis. We assessed the reliability and association with clinical symptoms of a method for assessing fatty infiltration, a simplified muscle fat index (MFI).

**Methods:**

Preoperative axial T2-weighted magnetic resonance imaging (MRI) scans of 243 patients aged 66.6 ± 8.5 years (mean ± standard deviation), 119 females (49%), with symptomatic lumbar spinal stenosis were assessed. Fatty infiltration was assessed using both the MFI and the Goutallier classification system (GCS). The MFI was calculated as the signal intensity of the psoas muscle divided by that of the multifidus and erector spinae. Observer reliability was assessed in 102 consecutive patients for three independent investigators by intraclass correlation coefficient (ICC) and 95% limits of agreement (LoA) for continuous variables and Gwet’s agreement coefficient (AC1) for categorical variables. Associations with patient-reported pain and disability were assessed using univariate and multivariate regression analyses.

**Results:**

Interobserver reliability was good for the MFI (ICC 0.79) and fair for the GCS (AC1 0.33). Intraobserver reliability was good or excellent for the MFI (ICC range 0.86–0.91) and moderate to almost perfect for the GCS (AC1 range 0.55–0.92). Mean interobserver differences of MFI measurements ranged from -0.09 to -0.04 (LoA -0.32 to 0.18). Adjusted for potential confounders, none of the disability or pain parameters was significantly associated with MFI or GCS.

**Conclusion:**

The proposed MFI demonstrated high observer reliability but was not associated with preoperative pain or disability.

## Key points

- Fatty infiltration of the paraspinal muscles can be quantified by magnetic resonance imaging (MRI).
- The proposed muscle fat index (MFI) uses routine lumbar MRI examinations.
- This novel MFI shows high observer reliability for the quantification of muscle fat.
- Increased muscle fat was seen in patients with lumbar spinal stenosis (LSS).
- Significant association between the MFI and symptoms of LSS was not found.

## Background

Degenerative lumbar spinal stenosis (LSS) is a clinical condition caused by degenerative changes in the supporting structures of the lumbar spine [1]. Patients with LSS experience varying degrees of disability, low back pain, and radiating pain in lower extremities [2]. Fatty infiltration of the paraspinal muscles is a frequent finding in patients with LSS [3, 4]. Mainly formed by the multifidus (MF) and the erector spinae (ES), these muscles are innervated by the dorsal rami of the L1–L4 nerves. The main function of the paraspinal muscles is extension and rotation of the lumbar spine and to resist gravity [5]. Studies have demonstrated associations between the severity of fatty infiltration of the paraspinal muscles evaluated by magnetic resonance imaging (MRI), and pain and disability reported by patients with LSS [3, 6, 7]. It has been suggested that fatty infiltration of the paraspinal muscles can be used as a predictor of postoperative clinical outcomes and recovery of patients with symptomatic LSS, influencing the treatment decision process [8–10].

Imaging modalities can be used for the assessment and grading of the severity of fatty infiltration in the skeletal muscles. The Goutallier classification system (GCS) is a frequently used semiquantitative grading method for the assessment of muscle fatty infiltration [11]. This method was originally proposed by Goutallier et al. [12] for grading the severity of fatty infiltration in the shoulder rotator cuff muscles on computed tomography (CT) as a prognostic tool for tendon repairs, suggesting a poorer outcome when the cuff muscles had higher fatty infiltration. Fuchs et al. [13] demonstrated good or excellent interobserver reliability for the GCS on shoulder CT and MRI individually, but only fair to moderate correlation between the GCS grading performed on CT and MRI. Despite this inferior correlation, the GCS has been adopted for the evaluation of muscular fatty infiltration on MRI in various anatomical locations, including the paraspinal muscles [14–17]. Both quantitative and semiquantitative MRI methods have been used to assess the severity of fatty infiltration in the paraspinal muscles. It has been suggested that quantitative MRI methods have higher reliability than the semiquantitative methods [18–20]. The main drawbacks of the currently available quantitative methods are time consumption and the need for exporting the images into a third-party software for analysis, making these methods less practical in everyday clinical practice [7, 19, 21, 22].

The muscle fat index (MFI) is a quantitative measure used by researchers to assess the fat content of the paraspinal muscles on MRI, by calculating the ratio of the mean signal intensity of the muscle of interest to a homogenous area of the same or another muscle [23]. In the current study, we introduced a new method for calculating the MFI based on the signal intensity of the paraspinal and the psoas major (PM) muscles measured on axial T2-weighted images from routine lumbar spine MRI examinations, without a need for using a third-party software. To our knowledge, this simplified method for calculation of the MFI has not been used earlier. We hypothesised that this easily accessible method might yield higher reliability than the GCS and, furthermore, would associate with the clinical symptoms. The purpose of this study was to evaluate the reliability of this novel MFI and assess its association with pain and disability in patients with LSS.

## Methods

### Study participants

**Fig. 1:**
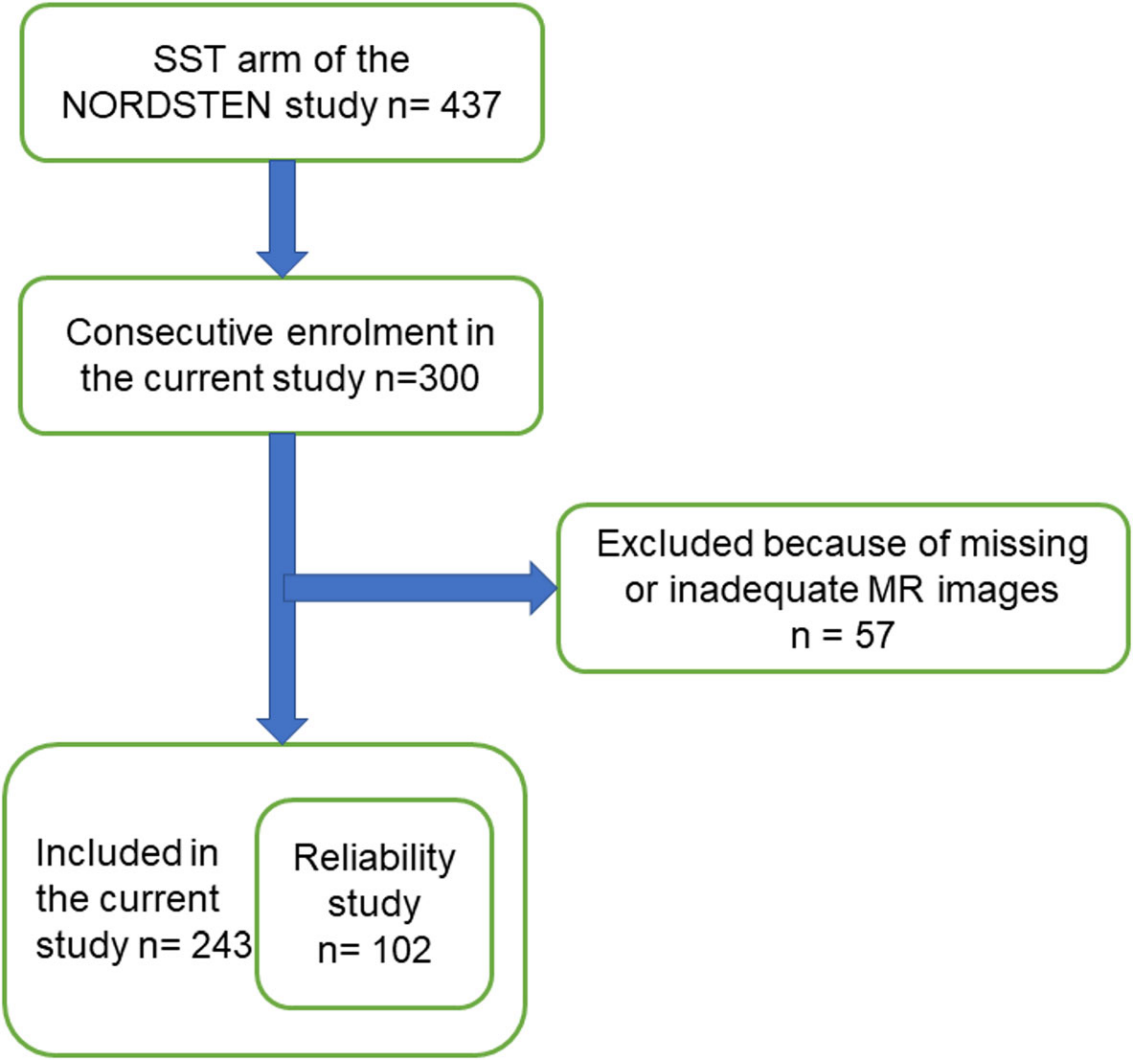
The flowchart shows the patient selection process. *SST* Spinal stenosis trial, NORDSTEN Norwegian degenerative spondylolisthesis and spinal stenosis

The regional committees for medical research ethics approved the current cross-sectional study (reference number: 2011/2034 central region). The study adhered to the Declaration of Helsinki and all patients provided written informed consent. The participants in this study were consecutively enrolled from the spinal stenosis trial of the Norwegian Spinal Stenosis and Degenerative Spondylolisthesis (NORDSTEN) study. This multicentre trial includes symptomatic patients with LSS without degenerative spondylolisthesis who are scheduled for surgery. The study protocol and the settings for inclusion and exclusion of the patients have been published earlier [24]. The inclusion and exclusion criteria for the current study are provided in Table 1. After the initial consecutive enrolment of 300 patients (convenient sampling based on the availability of patient data), we excluded 57 patients due to inadequate or missing images, leaving 243 patients who were finally included (Fig. 1).

**Table 1:** Inclusion and exclusion criteria

Inclusion criteria	Exclusion criteria
• Age between 18 and 80 years • Clinical symptoms of LSS • Not responding to at least 3 months of non-surgical treatment • Radiological findings (foraminal, central canal, or lateral recess stenosis) corresponding to the clinical symptoms such as back pain, leg pain, or neurologic claudication • Understanding the Norwegian language (spoken and written)	• Previous surgery at the level of stenosis • Previous fracture or fusion of the thoraco-lumbar spine • Cauda equina syndrome (bowel or bladder dysfunction) or fixed complete motor deficit • ASA grade 4 or 5 • More than 20° lumbosacral scoliosis • Distinct symptoms in lower limbs due to other diseases • Stenosis in more than three lumbar levels • Being unable to comply fully with the protocol • Isthmic defect in pars interarticularis at the level of stenosis • Participation in another clinical study that could interfere with the present trial • Alcohol or substance abuse • ≥ 3 mm spondylolisthesis verified on upright lateral view x-ray • Axial T2-weighted MR images not covering the paraspinal and the psoas muscles at both sides of the spine or angulated more than 5° to the upper endplate of the vertebra at the level of measurement

*ASA* American Society of Anesthesiologists, *LSS* Lumbar spinal stenosis

### MRI protocol and assessments

The preoperative MRI examinations used in this study were performed at the local study sites of the NORDSTEN study between February 2013 and August 2016 using 1.5-T or 3.0-T units from several manufacturers, with patients in supine position. All images were anonymised and stored in a dedicated server. To maintain homogeneity of the examinations, the performing institutions were provided with a standardised MRI protocol including axial and sagittal T2-weighted and sagittal T1-weighted images. A board-certified radiologist (H.B.) verified whether the qualities of the images were adequate for the present study (*e.g.*, the axial images covering both the paraspinal and the PM muscles on both sides of the spine). All measurements for the present study were performed on the axial T2-weighted images (repetition time 1,500–6,548 ms; echo time 82–126 ms; slice thickness 3–4 mm; field of view from 160 × 160 to 220 × 220 mm^2^).

**Fig. 2:**
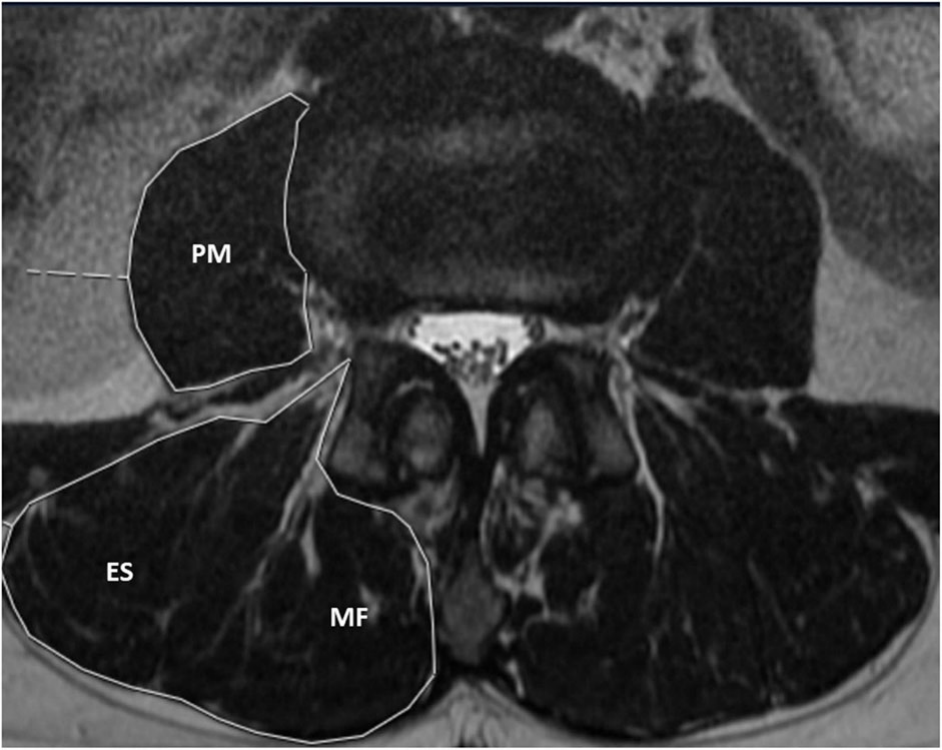
Axial T2-weighted magnetic resonance image obtained at the level of the upper endplate of L3. The muscle fat index (MFI) was calculated by dividing the mean signal intensity of the psoas major (PM) with the mean signal intensity of the erector spinae (ES) and the multifidus (MF) muscles

The paraspinal (ES and MF) and the PM muscles were evaluated bilaterally at the level with the upper endplates of L3, L4, and L5 (for both quantitative assessments of the MFI and semiquantitative assessments of the GCS). Inspired by previous studies [21, 23], the investigators segmented the paraspinal and the psoas muscles by drawing manual regions of interest around each muscle group. All segmentations were done using the integrated measurement tools in a Picture Archiving and Communication System (PACS) (Sectra, Linkoping, Sweden) on personal laptops with non-diagnostic monitors. The mean signal intensity of the MF and the ES muscles was measured by drawing a region of interest around both muscles, excluding the epimuscular fat. The signal intensity of the muscles for each region of interest was calculated automatically by the PACS. To assess the relationship between the fatty infiltration of the paraspinal and the PM muscles, we used the PM muscle as a natural control. It has been suggested that the PM muscle is less prone to fatty infiltration [3, 7, 25]. The MFI was calculated as a continuous variable by dividing the mean signal intensity of the PM with the mean signal intensity of the MF and ES on the same image slice and side. In this way, values close to 1.0 indicated near equal proportions of fat and muscle fibres in the paraspinal muscles compared to the PM, suggesting a very low degree of fatty infiltration; values close to zero suggested a very high degree of fatty infiltration in the paraspinal muscles. An example of this measurement method is shown in Fig. 2.

In the next stage (during the same session and on the same image slice used for calculation of the MFI), the severity of fatty infiltration was graded using the GCS as grade 0 (no fatty streaks), grade 1 (some fatty streaks), grade 2 (fatty infiltration but still more muscle fibres than fat), grade 3 (equal amounts of fat and muscle fibres), or grade 4 (larger amounts of fat than muscle fibres) [12].

### Assessment of observer reliability

Interobserver and intraobserver reliability for both methods were assessed for measurements performed at the levels from L2 to L5 for the first 102 consecutive patients. The investigators were three independent observers who were blinded to each other’s measurements and to the severity of pain and disability of the patients. They were two orthopaedic spine surgeons (E.H. and J.A. with 10 and 6 years of experience, respectively) and a musculoskeletal radiologist (H.B. with 13 years of experience in spine imaging). To assess the intraobserver reliability and to maintain the independency of the test-retest readings, all observers repeated the evaluations after a minimum of 6 weeks, blinded to the results of their first readings. Images with missing measurements or non-optimal axial T2-weighted images (*e.g.*, incomplete imaging of the muscles) were excluded and only levels with measurements from all the three observers were included in the reliability analyses. Prior to the study start, the investigators discussed the measurement criteria for both methods, and the segmentation method was presented to the orthopaedic spine surgeons by the radiologist. They performed test measurements of both the MFI and the GCS on 10 randomly chosen MRI examinations from the study population. The results of the test readings were not included in the statistical calculations.

### Assessment of clinical symptoms

Patient-reported outcome measures were used for clinical assessment of pain and disability, including:

- The Oswestry disability index (ODI) [26], a pain and disability index for use in low back pain ranging from 0 to 100, where 0 denotes no disability and 100 indicates complete disability
- The Zurich claudication questionnaire (ZCQ) for pain and disability [27], a disease-specific questionnaire for LSS with several sub-scores including the severity of the symptoms and level of physical activity, ranging from 1 to 5, where 1 indicates the best clinical outcome
- A numeric rating scale (NRS) for back and leg pain ranging from 0 to 10, where 0 indicates no pain and 10 indicates the worst pain imaginable [28]

### Statistical analyses

Continuous variables were described as means ± standard deviations and categorical variables as frequencies and percentages. Intraclass correlation coefficient (ICC) was calculated using two-way random effects models for absolute agreement and was used to assess the interobserver and intraobserver reliability for the MFI. Bland-Altman plots were used to assess the mean differences and 95% limits of agreements (LoA) for repeated measurements. The categorical ratings of the GCS were unevenly distributed, and thus, we used Gwet’s agreement coefficient (AC1) instead of *κ* statistics to assess the interobserver and intraobserver agreements (to avoid the so-called high agreement low kappa paradox) [29]. 95% confidence intervals (CIs) were calculated for both ICC and AC1. ICC values were interpreted to indicate poor (< 0.50), moderate (0.51–0.75), good (0.76–0.90), and excellent (> 0.91) agreement [30] and AC1 values to indicate poor (0.0), slight (0.01–0.20), fair (0.21–0.40), moderate (0.41–0.60), substantial (0.61–0.80), or almost perfect agreement (0.81–1.00) [31].

Observer 3 (radiologist H.B.) performed MFI measurements (continuous) and grading of the GCS (categorical) in the total study sample (243 patients). In few cases, the MFI values were higher than 1.0 and in the absence of apparent fatty infiltration in the PM, these values were redefined as 1.0. The measurements performed by observer 3 were used in the regression analyses and did not differ significantly between lumbar levels or sides (left/right). Thus, the values representing the highest fatty infiltration (lowest MFI or highest GCS values) from the L2/L3 level were entered into univariate and multivariate regression models, treating all the patient-reported outcome measures as continuous variables. Regression coefficients with corresponding 95% CIs were reported. In the multivariate regression models, we adjusted for age, sex, body mass index, and smoking status (yes or no). Because of the low prevalence of higher GCS grades and for better clinical relevance, we trichotomised the GCS values into category 0 (GCS grade 0, no fatty infiltration), category I (GCS grade 1, mild fatty infiltration), and category II (GCS grades 2 to 4, moderate or severe fatty infiltration) (Table 2). Model assumptions were assessed by normality plots of the standardised residuals and the fitted values. To compare the goodness of fit between the regression models, we calculated the Akaike information criterion (AIC). The AIC is a goodness of fit measure for comparing two models, where the regression model with the lowest AIC value fits better to the data. It has been suggested that an AIC difference of 2 to 7 should be considered as a meaningful difference between two models [32]; others have suggested a minimum difference of 6 AIC units [33]. Values of *p* lower than 0.05 were considered statistically significant. STATA software (StataCorp. LLC 2017. Stata Statistical Software: Release 16.1 College Station, TX, USA) was used for the statistical analyses.

**Table 2:** Patient characteristics and distribution of MRI findings

Patient characteristics (***n*** = 243)	Mean ± standard deviation/count (%)
Age (years)	66.6 ± 8.5
Female	119 (49)
BMI	27.8 ± 4.0
Smokers	55 (23)
DSCA < 75 mm^2^	
L2/L3 L3/L4 L4/L5	31 (19) 106 (44) 157 (65)
ODI score (100-point scale)	40.66 ± 14.57
NRS back pain (10-point scale)	6.28 ± 2.19
NRS leg pain (10-point scale)	6.38 ± 2.10
ZCQ pain (5-point scale)	3.38 ± 0.55
ZCQ disability (5-point scale)	2.58 ± 0.52
**MRI findings (L2/L3 level)**	
MFI*	0.53 ± 0.18
GCS	
Category 0 (grade 0) Category 1 (grade 1) Category 2 (grades 2, 3, and 4)	119 (49) 100 (41) 24 (10)

*BMI* Body mass index, *DSCA* Dural sac cross-sectional area, *GCS* Goutallier classification system, *MFI* Muscle fat index, *NRS* Numeric rating scale, *ODI* Oswestry disability index, *ZCQ* Zurich claudication score

*In one case, the value of the MFI was > 1.0, redefined as 1.0

## Results

### Patient characteristics

Patient characteristics and distribution of the MRI findings are presented in Table 2. The mean age was 66.6 years and 119 of the 243 included patients (49%) were women. The mean MFI value was 0.53, suggesting overall more than twice fat inside the paraspinal muscles compared to the PM. Most of the patients (*n =* 219, 90%) had GCS categories 0 or 1 (suggesting no or mild fatty infiltration in the paraspinal muscles) and 24/243 patients (10%) had moderate or severe fatty infiltration (categories 2 to 4). For the reliability part of the study (measurements at the L2–L5 levels), there were 424 GCS assessments and 418 MFI assessments per observer (not included in Table 2). There was an inverse relationship between the different grades of the GCS and the MFI values, indicating higher GCS grades in patients with lower MFI values (Table 3).

**Table 3:** Relationship between the MFI and the GCS

GCS grade	Mean MFI	95% CI
**0**	0.61	0.54, 0.68
**1**	0.42	0.39, 0.44
**2**	0.35	0.32, 0.37
**3**	0.30	0.26, 0.34
**4**	0.29	0.23, 0.35

*CI* Confidence interval, *GCS* Goutallier classification system, *MFI* Muscle fat index

### Interobserver and intraobserver reliability

The results of the reliability analyses are presented in Table 4. The agreement coefficients suggested good overall interobserver agreement for the MFI and only fair agreement for the GCS. Intraobserver agreement for the three observers was good or excellent for the MFI, while for the GCS, the agreement values ranged from moderate to almost perfect.

**Table 4:** Interobserver and intraobserver reliability

Reliability	MFI	GCS
	ICC (95% CI)	AC1 (95% CI)
**Interobserver**	0.79 (0.70, 0.85)	0.33 (0.27, 0.39)
**Intraobserver**		
Observer 1	0.91 (0.89, 0.92)	0.92 (0.89, 0.95)
Observer 2	0.86 (0.74, 0.91)	0.64 (0.59, 0.70)
Observer 3	0.91 (0.89, 0.93)	0.55 (0.49, 0.60)

*AC1* Gwet’s agreement coefficient, *CI* Confidence interval, *GCS* Goutallier classification system, *ICC* Intraclass correlation coefficient, *MFI* Muscle fat index

**Fig. 3:**
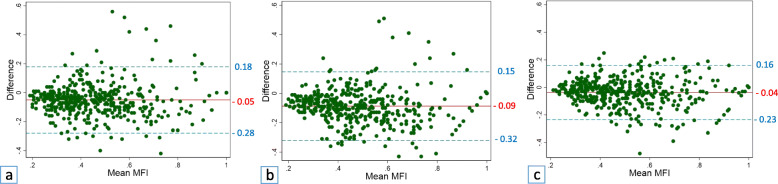
Bland-Altman plots with mean differences in measurements of the muscle fat index (MFI, solid lines) and 95% limits of agreement (dashed lines) between observers 1 and 2 (**a**), observers 1 and 3 (**b**), and observers 2 and 3 (**c**)

**Fig. 4:**
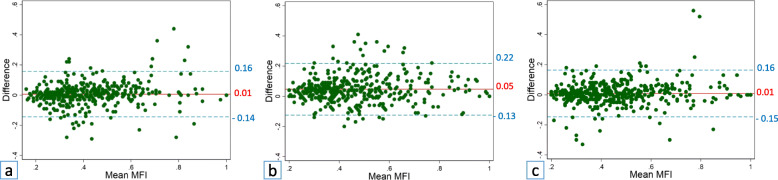
Bland-Altman plots with mean differences in measurements of the muscle fat index (MFI, solid lines) and 95% limits of agreement (dashed lines) for repeated measurements by observer 1 (**a**), observer 2 (**b**), and observer 3 (**c**)

Measurement differences for the MFI between all observer pairs, as well as within the observers, are demonstrated by Bland-Altman plots in Figs. 3 and 4, respectively. Mean interobserver differences (*i.e.*, mean bias) ranged from -0.09 to -0.04 with 95% LoA ranging from -0.32 to 0.18. The narrowest LoA for measurements (*i.e.*, the smallest measurement differences) were observed between observers 2 and 3 (one of the two surgeons and the radiologist, Fig. 3c).

Mean intraobserver differences ranged from 0.01 to 0.05 with 95% LoA ranging from -0.15 to 0.22. The narrowest LoA was achieved for observer 1 (one of the two surgeons, Fig. 4a).

### Association with clinical symptoms

The results of the univariate regression analyses are presented in Table 5. The estimated regression coefficients were generally small. We found a significant association only between NRS leg pain and the MFI (*p* = 0.042). A tendency towards lower AIC values was observed for the MFI (suggesting a better fitting to the univariate regression models of the MFI compared to the GCS).

**Table 5:** Univariate regression analyses

Clinical parameter	MFI			GCS*			
	***Coefficient (95% CI)***	***p***	***AIC***	***Category***	***Coefficient (95% CI)***	***p***	***AIC***
**ODI**	-6.90 (-17.38, 3.57)	0.195	1940	1	2.65 (-3.91, 9.21)	0.427	1945
				2	10.28 (-6.40, 26.96)	0.226	
**NRS leg pain**	-1.58 (-3.11, -0.06)	0.042	973	1	0.56 (-0.40, 1.52)	0.249	978
				2	0.66 (-1.72, 3.04)	0.584	
**NRS back pain**	-0.41 (-2.03, 1.21)	0.618	1011	1	0.86 (-0.15, 1.87)	0.093	1010
				2	1.48 (-1.02, 3.97)	0.245	
**ZCQ pain**	-0.30 (-0.71, 0.10)	0.136	388	1	0.06 (-0.20, 0.33)	0.629	394
				2	0.35 (-0.29, 0.99)	0.281	
**ZCQ disability**	-0.25 (-0.63, 0.13)	0.191	368	1	0.08 (-0.16, 0.32)	0.514	370
				2	0.51 (-0.09, 1.11)	0.098	

*AIC* Akaike information criterion, *CI* Confidence interval, *GCS* Goutallier classification system, *MFI* Muscle fat index, *NRS* Numeric rating scale, *ODI* Oswestry disability index, *ZCQ* Zurich claudication questionnaire

*For the GCS, only categories with fatty infiltration are presented. The coefficient values for the GCS show the estimates for the regression equation of categories 1 and 2, respectively, on category 0 (GCS grade 0, no fatty infiltration)

The results of the multivariate regression analyses are presented in Table 6. After adjusting for the potentially confounding factors, there were no significant associations between the patient-reported outcome measures and the MFI or the GCS. AIC values were consistently lower for the MFI and were 6 or 7 units lower in the analyses of the ODI and the ZCQ pain, suggesting better fitting of the MFI to the multivariate regression models.

**Table 6:** Multivariate regression analyses

Clinical parameter	MFI			GCS*			
	***Coefficient (95% CI)***	***p***	***AIC***	***Category***	***Coefficient (95% CI)***	***p***	***AIC***
**ODI**	-3.74 (-14.69, 7.21)	0.502	1863	1	0.10 (-5.69, 7.69)	0.769	1869
				2	7.06 (-0.26, 23.38)	0.395	
**NRS leg pain**	-0.59 (-2.13, 0.96)	0.455	913	1	0.21 (-0.70, 1.12)	0.649	916
				2	-0.23 (-244, 1.98)	0.837	
**NRS back pain**	0.80 (-0.84, 2.44)	0.339	950	1	0.63 (-0.34, 1.60)	0.199	952
				2	0.75 (-1.61, 3.10)	0.533	
**ZCQ pain**	-0.22 (-0.64, 0.20)	0.205	364	1	-0.01 (-0.26, 0.26)	0.996	371
				2	0.24 (-0.39, 0.86)	0.454	
**ZCQ disability**	-0.01 (-0.30, 0.03)	0.979	344	1	0.01 (-0.24, 0.25)	0.972	347
				2	0.40 (-0.19, 0.99)	0.186	

*AIC* Akaike information criterion, *CI* Confidence interval, *GCS* Goutallier classification system, *MFI* Muscle fat index, *NRS* Numeric rating scale, *ODI* Oswestry disability index, *ZCQ* Zurich claudication questionnaire

*For the GCS, only categories with fatty infiltration are presented. The coefficient values for the GCS show the estimates for the regression equation of categories 1 and 2, respectively, on category 0 (GCS grade 0, no fatty infiltration)

## Discussion

In this study, we found a high observer reliability for a novel quantitative MRI method (simplified MFI) in the assessment of fatty infiltration in the paraspinal muscles of patients with symptomatic LSS. For a more established semiquantitative method (the GCS), interobserver reliability was only fair and intraobserver reliability ranged from moderate to almost perfect. We found a significant association between leg pain and the MFI in the univariate regression analyses, but no significant associations in the multivariate analyses. However, the reliability coefficients and the AIC values suggested that the MFI presented here is a better fit to the regression models than the GCS.

Other quantitative methods have been used to assess the fatty infiltration of the paraspinal muscles on MRI. Researchers have used different software applications for texture analysis of the paraspinal muscles [22] or to assess the lean mass of the muscles by thresholding the signal intensity on MR images [7]. Both texture analysis and thresholding of the paraspinal muscles have shown high reliability [19, 21]. DIXON methods have gained increasing interest in spine imaging [34] and have been used for the quantification of fatty infiltration of the paraspinal muscles [35]. However, the need for exporting imaging data into a third-party software and performing additional MRI sequences makes these methods less practical in everyday practice. Whether the proposed MFI in the current study can be used on axial DIXON images of the lumbar spine needs further investigation.

Previous research has mainly focussed on the associations between back pain and fatty infiltration [6, 8, 22, 36–38]. Leg pain is a frequent symptom in patients with LSS and it is generally accepted that this symptom is a radiating pain from the lumbar spine [2]. There are, however, some controversies on whether this pain is solely generated by the lumbar nerves or can partly be caused by supporting structures of the spine [39, 40]. It has been suggested that patients with LSS and predominant leg pain are more likely to benefit from surgical decompression compared to those with predominant back pain [41]. We did not find a significant association between fatty infiltration of the paraspinal muscles and leg pain after adjusting for potential confounding factors. To our knowledge, this is the first study to examine this association in patients with LSS. Two studies not concerning LSS assessed leg pain in relation to fatty infiltration of the MF muscle with conflicting results. Fatty infiltration of the MF muscle was associated with leg pain in a retrospective study of 78 patients with low back pain [42] but not in a population-based cohort study of young individuals with a history of leg or back pain [43].

Several studies have examined the relationship between fatty infiltration of the paraspinal muscles and symptoms of degenerative diseases of the lumbar spine [6, 15, 23, 44, 45], but not all studies have considered the role of the PM muscle in this relationship [6, 15, 45]. To estimate the degree of fatty imbalance between the PM and the paraspinal muscles, we calculated the MFI by dividing the signal intensity of the PM with that of the MF and ES. The calculated mean MFI of 0.53 in the current study suggested overall less fatty infiltration in the PM compared to the MF and ES muscles, which is in accordance with previous research [3, 7, 25]. The important role of denervation in atrophy and fatty infiltration of the skeletal muscles have been demonstrated [46–48]. Higher fatty infiltration in the paraspinal muscles compared to the PM may support the role of damage of the dorsal rami of the lumbar nerves as a cause of fatty infiltration [49–51]. It is unclear whether nerve damage can be a common cause for fatty infiltration of the paraspinal muscles and leg pain in patients with LSS. However, it is important to be aware of this possible association in clinical practice. The MFI presented in this study provides a reliable and easy-to-perform quantitative method for assessment of fatty infiltration in the paraspinal muscles on a standard clinical MRI examination without a need of additional software resources and with a high potential to widespread use.

A limitation of this study was the highly symptomatic surgical sample, potentially leading to an underestimation of any association between fatty infiltration and symptoms (due to potential restriction of range) [52]. Furthermore, the results of this study are limited to patients with LSS. Another limitation that may have influenced the reliability was the heterogeneity of the MR images. Images obtained from different MRI units and manufacturers can differ in brightness, affecting the perception of the signal intensity. This may partly explain the lower reliability for the subjectively evaluated GCS in this study, but hardly affected the MFI measurements. We excluded the epimuscular fat of the paraspinal muscles in the MFI measurements; some studies have included this fat in quantitative measurements. There is, however, a lack of consensus on whether the epimuscular fat should be included or excluded from the measurements [20].

We did not measure time consumption in this study, but time is an important factor in clinical and radiological everyday practice. Quantitative MRI methods are generally more time-consuming compared to semiquantitative and qualitative methods [20]. The advent of artificial intelligence methods for automated segmentation of muscles and the integration of these methods with clinical PACS solutions are expected to resolve the time-consumption issue [53]. We used the signal intensity of the muscles for the assessment of fatty infiltration. It can be argued that the proportion of fat and muscle fibres (used in the GCS) can be applied in artificial intelligence methods to improve the assessment of fatty infiltration in the paraspinal muscles as well (*e.g.*, by calculating the lean muscle to fat ratio). Whether such method would result in better reliability and association with the clinical symptoms of patients with LSS is yet to be examined.

This simplified MFI method using routine MR images should be investigated in a broader patient population with LSS, also including patients without the need of surgical treatment, as well as to see whether fatty infiltration of the paraspinal muscles can be used as a predictor for postoperative outcomes of LSS.

In conclusion, the novel MFI proposed in this study presents a highly reliable method for the assessment of fatty infiltration in the paraspinal muscles using routine spine MRI examinations and measurement tools available in the PACS solutions. This MFI was not significantly associated with pain and disability in LSS but may provide better explanation for symptoms related to fatty infiltration in the paraspinal muscles, compared to the GCS.

## Data Availability

The datasets produced during this study are available from the corresponding author upon a reasonable request.

## Abbreviations

AIC: Akaike information criterion
CI: Confidence interval
CT: Computed tomography
ES: Erector spinae
GCS: Goutallier classification system
ICC: Intraclass correlation coefficient
LoA: Limits of agreements
LSS: Lumbar spinal stenosis
MF: Multifidus
MFI: Muscle fat index
MRI: Magnetic resonance imaging
NRS: Numeric rating scale
ODI: Oswestry disability index
PACS: Picture Archiving and Communication System
PM: Psoas major
ZCQ: Zurich claudication questionnaire
